# Evaluation of *Mycobacterium avium* subsp. *paratuberculosis* isocitrate lyase (*IcL*) and ABC transporter (*BacA*) knockout mutants as vaccine candidates

**DOI:** 10.3389/fcimb.2023.1149419

**Published:** 2023-03-30

**Authors:** Razieh Eshraghisamani, Rakel Arrazuria, Lucy Luo, Jeroen De Buck

**Affiliations:** Faculty of Veterinary Medicine, University of Calgary, Calgary, AB, Canada

**Keywords:** *Mycobacterium avium* subsp. *paratuberculosis*, live-attenuated vaccines, dairy calves, immune responses, control programs, vaccine development

## Abstract

There has been little success in controlling Johne’s disease, caused by *Mycobacterium avium* subsp. *paratuberculosis*, due to suboptimal diagnostics and the ineffectiveness of available vaccines. By knocking out *BacA* and *IcL*, genes required for MAP survival in dairy calves, two live-attenuated vaccine candidates were created. This study evaluated the host-specific attenuation of MAP *IcL* and *BacA* mutants in mouse and calf models, as well as the elicited immune responses. Deletion mutants were generated in MAP strain A1-157 through specialized transduction and found viable *in vitro*. First, the mutants’ attenuation and elicited cytokine secretion were assessed in a mouse model, 3 weeks after intraperitoneal inoculation with MAP strains. Later, vaccine strains were assessed in a natural host infection model where calves received 10^9^CFU oral dose of MAP wild-type or mutant strains at 2 weeks old. Transcription levels of cytokines in PBMCs were evaluated at 12-, 14-, and 16-weeks post-inoculation (WPI) and MAP colonization in tissue was assessed at 4.5 months after inoculation. Whereas both vaccine candidates colonized mouse tissues similarly to wild-type strain, both failed to persist in calf tissues. In either mouse or calf models, gene deletion did not reduce immunogenicity. Instead, inoculation with Δ*BacA* induced a greater upregulation of proinflammatory cytokines than Δ*IcL* and wild-type in both models and a greater expansion of cytotoxic and memory T-cells than uninfected control in calves. Δ*BacA* and wild-type strains significantly increased secretion of IP-10, MIG, TNFα, and RANTES in mice serum compared to uninfected control. This agreed with upregulation of IL-12, IL-17, and TNFα in calves inoculated with Δ*BacA* at all time points. The Δ*BacA* also gave rise to greater populations of CD4+CD45RO+, and CD8+ cells than uninfected control calves at 16 WPI. Low survival rate of MAP in macrophages co-incubated with PBMCs isolated from the Δ*BacA* group indicated that these cell populations are capable of killing MAP. Overall, the immune response elicited by Δ*BacA* is stronger compared to Δ*IcL* and it is maintained over two different models and over time in calves. Further investigation is warranted to evaluate the *BacA* mutant's protection against MAP infection as a live attenuated vaccine candidate.

## 1 Introduction

Paratuberculosis is a production-limiting enteritis in domestic (Behr et al., 2020) and feral ruminants (Pavlik et al., 2000) due to infection with *Mycobacterium avium* subsp. *paratuberculosis* (MAP). World dairy industries suffer from financial implications of Johne’s disease in cattle (Münster et al., 2013). Clinical manifestation of Johne’s disease includes persistent dysentery, emaciation, inadequate lactation, decreased generative capacity, and ultimately death due to loss of fluids and frailty. MAP is a phagosomal pathogen that spreads through ingestion of fecal matter or contaminated milk. MAP passes through the digestive system and primarily enters the sub-epithelial layer of the small intestine *via* the microfold (M) cells of Peyer’s patches, although it is possible for epithelial cells to also be involved in internalizing MAP (Momotani et al., 1988; Pott et al., 2009). MAP is then taken up by sub-epithelial macrophages through different receptor types: toll-like receptors, CR3, FcRs, and mannose-binding receptors, resulting in distinct patterns of cytokine secretion (Guirado et al., 2013). Once inside the macrophage, MAP inhibits phagosome maturation, phagolysosome formation, and phagosome acidification (Cheville et al., 2001; Hestvik et al., 2005), and interferes with macrophage apoptosis (Kabara et al., 2010). Therefore, macrophages intended to destroy MAP usually fail to do so and provide a protective shelter for MAP to survive, replicate, spread and infect other cells (Tessema et al., 2001).

Due to a limited understanding of Johne’s disease pathogenesis, immunology, and correlates of protection, it has been difficult to develop an effective vaccine to prevent the infection. Phagocytic cells are important for the innate immune response to this intracellular pathogen, but cellular immunity has key function in clearing the infection by recognizing and responding to specific antigens presented by phagocytic cells (Leite et al., 2015). A Th-1 type response is persistent during the early stages of the infection. While the host is actively producing IFN-gamma to activate and expand effector immune cells, MAP inhibits macrophage responsiveness to this cytokine (Arsenault et al., 2012; Arsenault et al., 2014). The secretion of IFNγ, IL-12, Il-18, CXCL9, and CXCL10 result in development of effector cells (Th1, Th17) from naïve T cells. The effector cells control the infection in initial stages but does not eliminate it. However, the secretion of IL-10 and TGFβ result in proliferation of regulatory T cells (Treg) and induction of anti-inflammatory responses. After cell-mediated immunity fails to eliminate MAP , there is a shift from type 1 response to ineffective antibody-mediate type 2 (Th-2) response, usually coinciding with disease progression (Stabel, 2000). Subversion of immune pathways by MAP in infected cells could be responsible for this change (de Almeida et al., 2008). In other words, a decline in cellular responses due to a dysfunctional immune response leads to transition to humoral immune responses, when cell-mediated responses are compromised (Begg et al., 2011). However, most recently, there has been some controversy about this immune type conversion paradigm as infected animals may experience a substantial overlap of type 1 and type 2 immunity (Stabel and Bannantine, 2019).

Current control strategies focus on preventing the transmission of MAP through management practices (Barkema et al., 2018) by testing and culling positive animals, decreasing the incidence of new infections, preventing spread, and protecting negative herds, and decreasing prevalence in bulk tank milk (Wolf et al., 2014). Identifying and selectively breeding cattle with natural resistance to MAP infection is also an approach recently employed by Canada and some European countries (Alonso-Hearn et al., 2022; Sanchez et al., 2022). Despite considerable efforts to control Johne's disease, their effectiveness has been limited mostly due to high costs, suboptimal diagnostics, and incomplete knowledge of MAP pathogenesis (Nielsen and Toft, 2006; Nielsen and Toft, 2008). Paratuberculosis control in small domestic ruminants in Australia is primarily achieved using a killed vaccine (Gudair^TM^, Zoetis, Australia) (Reddacliff et al., 2006). This vaccine is also commercialized and used against paratuberculosis in sheep and goat in some European countries like France and Spain. Currently available cattle vaccines, that are mostly based on inactivated whole bacteria, have shown to slow disease progression (Juste et al., 2009) and provide therapeutic benefits by reducing bacterial burden in both tissue and fecal samples of infected adult cows (Alonso-Hearn et al., 2012; Juste et al., 2016a). However, they have been impeded by their deficiencies, including interference with tuberculosis diagnostics, limited prevention of MAP spread, and incapability in stopping infection establishment in tissue (Kalis et al., 2001; Stabel et al., 2011). Diverse approaches have been attempted to develop an effective paratuberculosis vaccine that prevents the establishment of a persistent infection as vaccination could be a cost effective control method (Bastida and Juste, 2011). Recently, interest in developing Johne's disease vaccines based on genetically attenuated mutants has grown. This approach has been useful for some other intracellular bacterial pathogens including *S.* typhi (Booth et al., 2020) and *M.* tuberculosis (Andersen and Scriba, 2019). A global regulator, relA, was removed from the MAP genome in one example of live-attenuated JD vaccine candidate. *In vitro* and *in vivo* experiments involving the infection of bovine macrophages, goat and calf infection model indicated a significant reduction in relA mutant survival compared to wild-type controls. The Δ*relA* was attenuated for survival in calf and goat tissue and it also decreased the capacity of the infection strain to colonize in tissue, evident by reduced number of positive tissue sites in vaccinated animals (Park et al., 2011). Further investigation indicated that this vaccine strain can induce the formation of cytotoxic T lymphocytes that can eliminate MAP (Abdellrazeq et al., 2018). Vaccination with pgsN mutant also induced strong cell-mediated immune responses, reduced bacterial burden in tissue and fecal shedding in challenged goats and calves (Shippy et al., 2017; Phanse et al., 2020). While mycobacterial live attenuated vaccines can be effective in providing protection against infection, they are not without limitations. Potential limitations are the prolonged persistence of the vaccine in the body, interference with diagnostics, and the persistence of the induced immune responses (Flores-Valdez et al., 2022). While this type of vaccine against intracellular infections could provide long-lasting cell-mediated immune responses, it may not induce the response as quickly as other types of vaccines. It is important to note that these limitations do not necessarily outweigh the benefits of this type of vaccine, but they do need to be taken into consideration when designing vaccination programs and evaluating their effectiveness.

**Figure 1:**
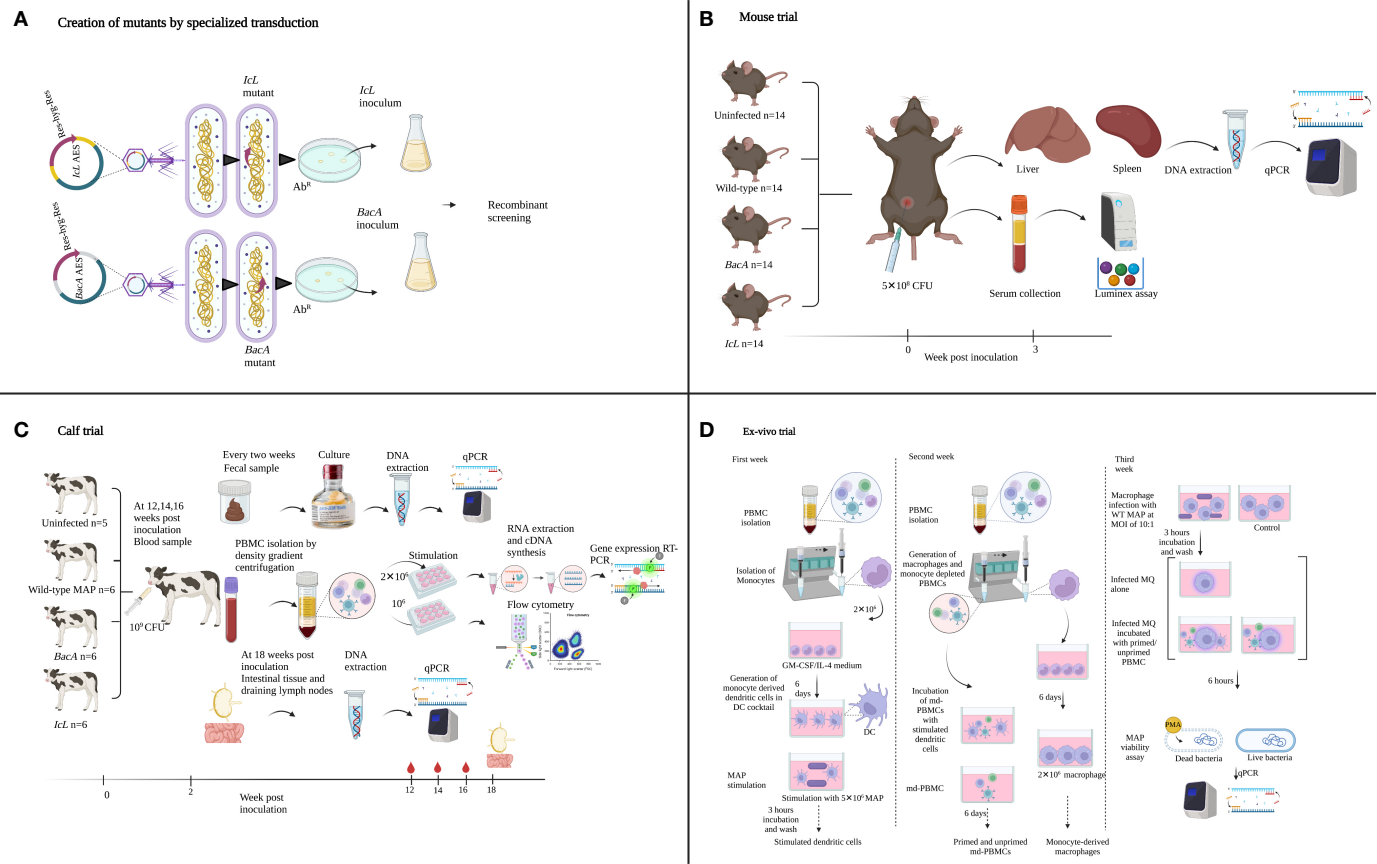
Summary of the experimental methods used to create and evaluate MAP IcL and BacA mutants in mouse, calf, and ex-vivo trials. Illustrations were created in Biorender.

Screening a library of MAP transposon mutants in calf infection model categorized MAP coding regions into 683 essential, 304 growth-defect, 3277 non-essential and 92 growth-advantage coding regions (EshraghiSamani et al., 2022). Allelic exchange mutagenesis was employed to create two MAP live attenuated strains by knocking out one essential gene (*IcL*, MAP1643) and one growth-defect gene (*BacA*, MAP1531c) (**Figure 1A**). The essential genes are identified to be necessary for MAP growth *in vivo* and the absence of growth-defect genes is expected to result in impaired growth of MAP. MAP1643 encodes an isocitrate lyase, an essential enzyme that mediates the cleavage of isocitrate to succinate and glyoxylate, a part of glyoxylate shunt or the tricarboxylic acid cycle (TCA cycle). The glyoxylate shunt is required for intracellular bacteria at the latent stage when fatty acids are considered the main source of carbon (Serafini et al., 2019). The requirement of this gene depends on the status of immune cells. The growth of *M. tuberculosis* Δ*IcL* decreased negligibly in resting macrophages, but the rate of growth was markedly low in activated macrophages (McKinney et al., 2000). MAP1531c encodes BacA, an ATP-binding cassette transporter involved in the acquisition of hydrophilic compounds like vitamin B12. With the help of irtAB, an ATP-binding cassette transporter involved in siderophore acquisition, BacA also plays a role in iron metabolism and enables MAP survival in the iron-limiting environment of the phagosome. The *bacA* orthologous gene in *M. tuberculosis* is required for the maintenance of chronic infection (Domenech et al., 2009) and is less involved in infection establishment.

In the present study, these two newly developed live-attenuated vaccine candidates were first screened in a mouse model to evaluate the species-specific essentially of selected genes, and to evaluate the production of Th1 type cytokines (**Figure 1B**). Next, the attenuation in calf was confirmed and the capacity of these candidate vaccines to induce immune responses in the natural host was evaluated in the calf model (**Figure 1C**). To further evaluate the functionality of effector cells in response to vaccine candidates, an *ex-vivo* viability assay was employed (**Figure 1D**).

## 2 Material and methods

### 2.1 Bacterial strains and growth conditions

Bacteria growth conditions were previously explained (Luo, 2018). Shortly, the *Escherichia coli* strains DH5α (New England BioLabs (NEB)), HB101 (Bio-Rad, Hercules, CA, USA) and ClearColi® BL21 (Lucigen, Middleton, WI, USA) and fast-growing mycobacteria *Mycobacterium smegmatis* mc (Pavlik et al., 2000)155 (ATCC, Manassas, VA, USA), were grown in Luria-Bertani (LB) Miller broth (Invitrogen, Carlsbad, CA, USA) at 37°C. LB Lennox agar plates (Invitrogen) with 100 µg/ml hygromycin (Sigma-Aldrich Canada Co., Oakville, ON, Canada) were used to pick successfully transformed *E. coli and M. smegmatis* . Difco Middlebrook 7H9 media (Becton Dickinson and Company (BD), Sparks, MD, USA) enriched with 10% OADC (Oleic Albumin Dextrose Catalase; BD), 2 mg/L mycobactin J (Allied Monitor Inc., Fayette, MO, USA) and 0.4% glycerol (Fisher Scientific, Fair Lawn, NJ, USA) was used to culture MAP A1-157, which is associated to a major clade accounting for over 80% of all MAP isolates found in Canada (Ahlstrom et al., 2015). Transduction happened at 30°C, the permissive temperature for the phasmid to inject its DNA into the bacteria. Transductants that received the hygromycin resistance gene were picked on Difco Middlebrook 7H11 plates (BD) with 75 µg/ml hygromycin and grown at 37°C with shaking at 225 rpm.

### 2.2 Creation of MAP mutants

#### 2.2.1 Construction of allelic exchange substrates

Cosmid pYUB854 (kindly supplied by the Jacobs’ lab, Albert Einstein College of Medicine) was employed to generate the allelic exchange substrates (AESs), as previously described (Luo, 2018). This cosmid possess a selectable resistance cassette (resolvase-hygromycin-resolvase) surrounded by multiple cloning sites. To target essential genes, primer pairs were crafted with two unique restriction enzymes sites, two on the upstream (L arm) and two on the downstream (R arm) of each gene. The specific restriction enzymes used for the L arm were StuI and XbaI, and for the L arm were HindIII and SpeI (NEB). The flanking arms were 800 bp in length and extended a 50 bp homologous sequence on both upstream and downstream of the targeted essential gene sequence. Utilizing MAP A1-157 DNA, the flanking arms were created by PCR with the following settings: 1.5 min of pre-denaturation at 95°C followed by 35 cycles of denaturation at 95°C for 30 s, 30 s of annealing at 60°C, extension at 72°C at 1 kb/min, and a 5 min final extension at 72°C. The ligation procedure was performed using a 1:2 ratio of insert DNA to vector DNA and left to incubate at room temperature for an entire night. The L arm was inserted into pYUB854 first, and then the R arm. The successful insertion was verified through sequencing.

#### 2.2.2 Creating the specialized transducing phage

Temperature-sensitive shuttle phasmid phAE159 (45 kb), which contain an ampicillin resistance cassette and replicates as a lytic phage at 30°C, was employed to generate the specialized transducing mycobacteriophage (Luo, 2018). The shuttle phasmid and allelic exchange substrates DNA were concentrated three times by lypholization, resulting in concentration of 150 ng/μl and 500 ng/μl, respectively. The phAE159 and AES DNA were combined at a 1:1 ratio after being digested with PacI enzyme. The process of digesting phAE159 with PacI enzyme resulted in the elimination of the ampicillin resistance cassette, making the resulting ligated product resistant to hygromycin due to the presence of a hygromycin resistance cassette in AES. The in vitro packaging (Gigapack XL, Agilent, Santa Clara, CA, USA) was done via the lambda cos site in the AES. E. coli strain HB101 was transduced with the specialized transducing phage and picked for hygromycin resistance. The absence of resolvase function in HB101 strain (Jain et al., 2014) garantees the intactness of AESs. QIAprep Spin Miniprep Kit (Qiagen) was utilized to extract and purify the 51 kb long phasmid DNA. Both phasmid DNA and AESs were sequenced to confirm the recombination and intactness of left and right arms, respiectively. Fast-growing M. smegmatis was used to amplify the mycobacteriophage as follows: transformation of 600 ng of the phasmid DNA into 400 μl of electrocompetent M. smegmatis at 30°C using the settings 2500 V, 25 μF, 1000 Ω , incubation of cells with 1 ml of LB broth at 37°C for 1 h, acquiring the phage plaques after 3 days after spreading the mixture on on room temperature LB plates and incubated at 30°C. Each single plaque was extracted from agar using a inoculation loop, and dispersed into mycobacteriophage buffer (MP buffer: 50 mM Tris-HCl pH 8.0, 150 mM NaCl, 10 mM MgCl_2_, 2 mM CaCl_2_). Extracted phages were then incubated with M. smegmatis, and caltivated on LB agar plates at 30°C for 2 days. To collect the amplified phages, MP buffer was added to the plates gentely shaking on a rocker for 30 minutes at room temprature. The bacterial lawn containing phages was harvested into 50 ml conical tubes, spined down at 4000 x g for 20 min. The clear supernatant was passed through a 0.45 μm filter. The phage stock was titerated by inoculation M. smegmatis (OD_600_ = 0.8-1.0 ) with 10^-8^ and 10^-9^ dilutions at 30°C. Control plates were also incubated at 37°C to affirm the temperature-sensitivness of STP.

#### 2.2.3 Specialized transduction

STP act as a lytic phage at 30°C, and as a DNA injecting vector at 37°C. So, the specialized transduction and following incubations were carried out at 37°C, to let the temperature sensitive phage inject its DNA into MAP cells without causing cell lysis. The specialized transduction phage stoke was pre-incubated at 37°C while MAP cultures were being prepared for transduction as follows: a 10 ml culture of MAP A1-157 strain was grown to an OD_600_ = 0.6-0.8 at 37°C, 140 rpm, the cultures were then subcultured at a 1:10 ratio into 7H9 media. The cells were again grown to an OD_600_ = 0.6, collected and washed with MP buffer at 4000 rpm for 10 min at RT. The resulting pellet were transduction-competent MAP cells resuspended in 500 μl of MP buffer. The transduction-competent MAP cells were incubated with STP at a multiplicity of infection (MOI) of 10 and incubated at 37°C. After 20 hours, the transduction product was centrifuged, and the pellet was resuspended in 200 μl of 7H9 media. The transduced MAP cells were plated on 75 μg/ml hygromycin-7H11 plates and incubated at 37°C for 4-6 weeks to select colonies that had successfully accomplished homologous recombination (**Figure 1A**).

#### 2.2.4 Homologous recombination screening

**Figure 2:**
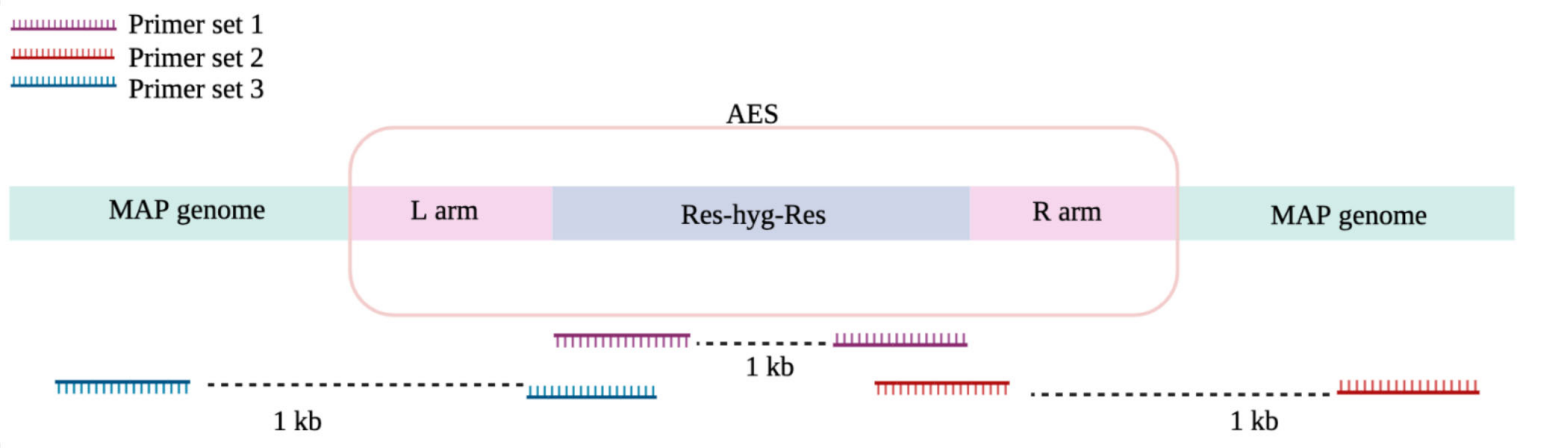
PCR primer schematic for MAP recombination screening.

Before proceeding with *in vitro* and *in vivo* experiments, the gene knockouts were confirmed. by screening for homologous recombination using PCR, as previously explained (Luo, 2018). The old school boiling method was employed to extract MAP genomic DNA as follows: single colonies from the plate were collected and dissolved in 800 μl pure ethanol and spun, and the resulting pellet was washed twice with 900 μl sterile Dulbecco’s Phosphate Buffered Saline (DPBS; Gibco), then simmer in 100 μl of sterile UltraPure DNase/RNase-Free Distilled Water (Gibco) for 30 minutes. After a final spinning, the supernatant was collected, and the DNA concentration and purity were evaluated *via* a NanoVue Plus (GE Healthcare Life Sciences). All centrifugations were performed at 7500 rpm for 9 minutes. Each PCR reaction was composed of TopTaq DNA polymerase (Qiagen), 3% DMSO and 200 ng of extracted DNA. The PCR protocol consisted of: a pre-heating step at 95°C for 1.5 min, 35 cycles of heating at 95°C for 30 seconds, cooling at 64°C for 30 seconds, elongation at 72°C at a rate of 1 kb/min, and a final elongation step of 5 minutes at 72°C. Three primer sets (**Table 1**) were designed to evaluate successful transduction, targeting the hygromycin resistance cassette (**Figure 2**; primer set1), the right arm crossover regions (**Figure 2**; primer sets 2), and the left arm crossover regions (**Figure 2**; primer sets 3). STP DNA and wild-type MAP DNA were used as positive and negative controls, respectively. Successful mutants that amplified all hygromycin resistance cassette, left arm crossover region, and right arm crossover regions were plated on a fresh 7H11 plate containing 75 μg/ml hygromycin. After 6 weeks incubation at 37°C, single colonies were selected to inoculate a fresh liquid culture. After 6 weeks incubation at 37°C and 215 rpm, the OD600 reached to 0.8-1 and the wet weigh method was used to quantify 5×10^8^and 10^9^ CFU of each strain for mice and calf trial, respectively. The mutants did not indicate any evidence of growth deficiency *in vitro*. The standard infection dose (Hines et al., 2007) for oral administration range from 5×10^6^ to 10^7^. The dose in present study was raised to 10 (Kabara et al., 2010), to prevent the risk of mutants’ disappearance from tissue due to low number of bacteria. Evaluation of the dose-dependency of immune responses and MAP persistence in tissue and fecal samples in experimental oral infections indicated that the higher dose of MAP (5×10^9^) indicated more consistent evidence of infection, compared to the lower dose (5×10^7^) (Mortier et al., 2014).

**Table 1:** Primers for screening MAP recombination.

Primer Name	Primer Sequence
Hygromycin_F_ps1	CGAGAACGTCCCCGACGTG
Hygromycin _R_ps1	GCGCCAGTTCCTCCGGATC
AES_F_ps2	CTCGAGACGTACGGTGCGCGCG
MAP_R_ps2	GTCGCTAATCGGCGCCTGCC
MAP_F_ps3	CAACGTCAGGAGTGTGGATG
AES_R_ps3	GCAACACCTTCTTCACGAGCAGAC

### 2.3 Mouse trial

#### 2.3.1 Mice

Fifty-six female 6-8 weeks C57BL/6 mice were acquired from Jackson Laboratories (Bar Harbor, ME, USA). All mice experiments were conducted under protocols that had been previously reviewed and approved by the University of Calgary's Veterinary Sciences Animal Care Committee (VSACC) with the reference number AC17-0220.Various groups of mice (n=14) were intraperitoneally inoculated. A control group was inoculated with PBS while the infection groups received 5x10 (8 )CFU of MAP mutants or wild-type strains (**Figure 1B**).

#### 2.3.2 Evaluation of MAP colonization in mice tissue

Liver and spleen samples were collected from each mouse 3 weeks after inoculation. Intraperitoneal injection of mice results in uptake of infection mostly by internal organs including liver and spleen. Samples were processed within 4 h employed for 1g tissue homogenization in 1 ml of 0.5% triton X100 PBS solution. The supernatant was placed in tubes with 1mm zirconia beads and homogenized in the bead beater. 0.2 ml of the supernatant was collected for nucleic acid isolation using the DNeasy® Blood and Tissue kit (Qiagen, USA) according to manufacturer’s instructions for Gram-positive bacteria. qPCR for identification of MAP DNA from collected tissue was performed as detailed below (section IX).

#### 2.3.3 Luminex assay

Blood samples were collected from mice before euthanasia. One ml of harvested serum was used in the Luminex assay, targeting eleven cytokines including IFNγ, IL-1β, IL-6, IL-10, IP-10, KC, MCP-1, MIG, MIP-1β, RANTES, and TNFα. Immune Monitoring 48-Plex Mouse ProcartaPlex^TM^ Panel (Invitrogen) was employed as directed by the manufacturer. Shortly, 50 μl of capture bead mix was added to each well of the plate. Before adding 25μl of Universal Assay Buffer (UAB) to each well, beads were washed twice using a hand-held magnetic plate washer. Then, 25 μl of samples, standards and controls were added to the plate. The plate was shacked at 600 rpm for two hours at room temperature before being washed twice. Using a multichannel pipette, 25 μl of the detection antibody solution was added to each well and shacked at 600 rpm for 30 minutes at room temperature. The plate was washed before and after adding 50 μl of Streptavidin-PE to each well. Then, 120 μl of reading buffer was added to the plate and the plate was run on Luminex^TM^ 200^TM^ Instrument (Invitrogen).

### 2.4 Calf infection trial

#### 2.4.1 Calves

Twenty-three Holstein-Friesian bull calves were collected within 12 hours after birth from Alberta dairy farms with a low prevalence of JD. The prevalence was assessed on environmental samples evaluated for MAP presence *via* quantitative polymerase chain reaction (qPCR) amplifying IS900 and F57 genes. Custom-built individual housing units were set up in a biosecurity level 2 research barn of the Veterinary Science Research Station (VSRS). Standard Operating Procedures were carried out in compliance with Veterinary Sciences Animal Care Committee (VSACC) protocol AC18-0059. Calves were randomized over four groups, including 5 uninfected healthy controls, 6 calves infected with WT field strain, 6 calves inoculated with *IcL* mutant strain, and 6 calves inoculated with *BacA* mutant strain. Vaccinated and infected groups were orally given an inoculation of 10 (9 )CFU of MAP strains in two sequential days at two weeks of age (**Figure 1C**). Animals received MAP strains in 7H9 Middlebrook without adding any adjuvant. The uninfected control group received plain 7H9 Middlebrook as sham inoculation.

#### 2.4.2 Isolation of blood peripheral mononuclear cells and cell culture

Blood was collected at 12-, 14-, and 16-weeks post-inoculation. Density gradient centrifugation was employed to separate peripheral blood mononuclear cells (PBMCs). Cells were seeded in a 24-well plate at a density of 2x106 cells/ml in a complete medium consisting of RPMI 1640 (Gibco, Grand Island, NY), 10% fetal calf serum (Atlanta Biologics, Atlanta, GA), 100 U/ml of penicillin G sodium (Gibco), 100 μg/ml of streptomycin sulphate (Gibco), 0.25 μg/ml of amphotericin B (Gibco), and 2 mM L-glutamine (Gibco).The following *in vitro* treatments were conducted in triplicate wells for each animal: PBS (negative control), pokeweed mitogen (10 μg/ml; Sigma), and avium PPD (10 μg/ml; Thermo Scientific). For gene expression analysis, plates were removed at 24 h and cells were harvested for RNA extraction. Cells were also harvested for flow cytometric analysis from a replicate set of plates.

#### 2.4.3 Flow cytometric analysis of stimulated PBMCs

Stimulated and unstimulated PBMCs were labelled with a mixture of four conjugated surface antibodies including CD4/MaxLight405 (Cat#227418-ML405, USBiological Life Science, USA), CD8/Maxlight490 (Cat#214734-ML405, USBiological Life Science, USA), CD45RO/AlexaFlour647 (Cat# MCA2434A647, Bio-rad), and WC1/FITC (Cat# MA5-28518, Thermofisher) diluted in FACS buffer. Cells were then incubated for 30 minutes at RT. After a wash step, cells were incubated with fixation/permeabilization concentrate for 30 minutes to fix the surface and permeabilize cells for intracellular staining. Cells were then labelled for intracellular marker FOXP3/ AlexaFlour680 (Cat# HCA116A647, Bio-rad) antibody, diluted in permeabilization buffer. Data were collected with Invitrogen Attune NXT software from an Attune NXT cytometer and analyzed with FlowJo software (BD, Canada). Mononuclear leukocytes, identified based on forward- and side-scatter size properties, were evaluated for cell surface markers CD4, CD8, WC1, and CD45RO and intracellular markers including FOXP3 expression. Different subpopulations were expressed as percentages of mononuclear leukocytes. All the antibodies were bovine specific conjugated antibodies.

#### 2.4.4 Relative quantification of cytokines expression

After 24 hours of *in vitro* stimulation RNA was extracted from PBMCs. After centrifuging the plates for 5 minutes at 1,500 rpm, duplicate wells were harvested and lysed with 350 μl of RLT buffer. Following the manufacturer's instructions, RNA was isolated using RNeasy minikit (Qiagen) and eluted with 30 μl of TE buffer (Ambion, Austin, TX). RNA (300 ng) and reverse transcribed with SuperScript III (Invitrogen, Carlsbad, CA) using 150 ng of random hexamers, 10 mM deoxynucleoside triphosphates (dNTPs), and 40 U of RNaseOut (Invitrogen). After being heated to 65°C for 5 minutes, samples were reverse transcribed for 60 minutes at 50°C. The cDNA was stored at -80°C prior to use in RT-PCR. 

Real-time PCR was conducted utilizing TaqMan gene expression assays for bovine IL-4, IL-10, TGFB, IL-12, IL-17A, IL-18, IFNG, IP10, and MIG (Life Technologies, Grand Island, NY) as directed by the manufacturer. All TaqMan primers were designed for amplifying exon-exon junction. A 20 μl reaction mixture including TaqMan universal PCR master mix, forward and reverse primers, and a 6-carboxyfluorescein (FAM)-MGB probe was prepared using 2 μl cDNA template. The mRNA levels of genes of interest were normalized using a eukaryotic 18S rRNA endogenous gene expression control (FAM-MGB probe, non-prime limited; Invitrogen). To compare the level of gene transcription in PBMCs after stimulation with 10 μg/ml MAP antigen (PPDa) (BOVIGAM™ Tuberculin PPD Stimulating Antigen, avian), the non-stimulated cells were used as a calibrator and all the upregulation and downregulation of the genes in stimulated cells are compared to the non-stimulated homolog of the same sample. The data were analyzed with the 2^-ΔΔct^ method. Shortly, Δct of each gene was calculated based on the ct value of the housekeeping gene (18S rRNA) and the ΔΔct was calculated applying the unstimulated control cells as a calibrator. The results were presented as a relative gene transcription compared to the unstimulated control.

#### 2.4.5 Fecal sample culture

 Fecal samples were cultured with TREK para-JEM culture media (TREK Diagnostic Systems, Cleveland, OH, USA) followed by F57 and IS900 quantitative polymerase chain reaction (qPCR), as explained previously (Slana et al., 2008; Corbett et al., 2018). Briefly, 2 g of feces was dissolved in 30 ml of autoclaved distilled water in a 50 ml conical tube and incubated for 30 minutes at RT. To remove any potential microbe other than MAP, 5 ml of supernatant was mixed with a 25 ml solution of 0.9% hexadecylpyridinium chloride (HPC; Alfa Aesar, Heysham, England) and half-strength brain health infusion (BHI) solution. After 24 hours of incubation at 37°C, samples were centrifuged at 3700 × *g *for 20 min. The pellet was mixed with antibiotic solution (AS; Para-JEM), water, and full-strength BHI, and incubated for a day at 37°C. Then, 1 ml of the supernatant was injected to liquid culture medium in TREK para-JEM culture bottles and incubated at 37°C for 49 days at 37°C.

#### 2.4.6 Intestinal tissue sample processing and culture

Calves were sacrificed 18 weeks after inoculation. The calves were first sedated with xylazine (0.3 mg/kg) and then received intravenous injection of barbiturate (Sodium pentobarbital; trade name Euthanyl Forte®, DIN 00241326, Bimeda-MTC Animal Health Inc., ON, Canada). Six type of tissue samples were obtained from each calf: ileum, distal jejunum, ileocaecal valve, ileal lymph nodes, distal jejunal lymph nodes, and ileocaecal valve lymph nodes. Cross-contamination was prevented by using sterile tools and fresh gloves for each tissue sample. To prevent the movement of intestinal contents, zip ties were used to mark and isolate sample locations before collection. The lymph nodes were collected first, before any examination of the intestinal tissue was conducted. After being collected, the lymph nodes were cleaned using PBS solution, while the intestinal tissue was cleaned by rinsing it with water. This is done to ensure accurate analysis by removing any contaminants from the samples.

Within 4 hours of collection, samples were processed as previously stated (Mortier et al., 2013). To sample the mucosal layer of the intestinal tissue microscope slides were employed. Lymph nodes (LNs) were diced using new scalpel blades between samples after removing the fat. GentleMACS M tubes were used to homogenize a total of 2.5 g of each sample in 10 ml of a PBS solution containing 0.5% triton X-100. The dissociation parameters were 2753 rounds per run for 53 s (rpr), and 3 runs per sample. Following the transfer of the samples into a 50 ml falcon tube, the samples were centrifuged at 3700×g for 20 min. The pellet was then mixed with 25 ml of 0.75% HPC and half-strength BHI with 4-mm sterile glass beads (n = 10) and vortexed vigorously for 2-3 min. Following a 3-hour incubation period at 37°C, samples underwent 15 min centrifugation at 3700 × g. After being mixed with 3 ml of antibiotic brew (0.2 ml paraJEM® AS (Vancomycin, Nalidixic Acid, and Amphotericin), 1.5 ml full-strength BHI, and 1.3 ml ddH2O), the pellet was incubated at 37 °C for an overnight period before 1 ml was transferred to paraJEM® culture bottles and cultured for 49 days. 

#### 2.4.7 DNA extraction from culture

Following the 49-day incubation, the same approach was used to process the tissue and fecal cultures. 200 μl of culture broth was combined with 4 volumes of pure ethanol, then centrifuged at 7500 rpm for 9 min. The resulting pellet was washed with Dulbecco’s Phosphate Buffered Saline (DPBS; Gibco, Carlsbad, CA, USA), then heated to the boiling point in 100 μl of sterile UltraPure DNase/RNase-Free Distilled Water (Gibco) for 30 min. The supernatant was isolated after a final centrifugation stage at 7500 rpm for 2.5 min and stored for use in qPCR. DNA extraction negative and positive controls were also included.

#### 2.4.8 DNA extraction from homogenized tissue

Total DNA was extracted from intestinal mucosa and lymph nodes as previously described (Sivakumar et al., 2005). Briefly, one gram of tissue was dissociated in 1 ml of TE buffer (10 mM TRIS-chloride, 1 mM EDTA, pH 8.0), using GentleMACS M tubes and Miltenyi dissociator, and incubated for 30 min. Supernatant were moved to a clean Eppendorf tube. The supernatant was then mixed with Lysozyme (5 mg/ml, Sigma, MO, USA) and kept at 37 °C for 2 hours. After adding SDS (1mg/ml) and proteinase K (2mg/ml) at, tubes were kept in heating block at 56°C overnight. After enzyme digestion, 0.4 volume of 5 M potassium acetate was added, samples were placed on ice for 10 minutes, and centrifuged at 9600×g for 12 (It should be (Cross, ×) not (x)). Phenolic extraction was employed to purify DNA from the supernatant as follows: tubes were inverted 20 times after adding one volume of tris-saturated phenol: chloroform: isoamyl alcohol (25:24:1) (Sigma) to the supernatant. The aqueous phase was isolated after centrifuging the tubes at 9600 ×g for 12 min. To precipitate the DNA, 1/10 volume of cold 3 M sodium acetate and 2.5 volumes of pure ethanol were added to the aqueous phase. The harvested DNA pellet was washed with 70% ethanol and reconstituted in 20 μl TE buffer. Five aliquots of DNA extracted from each tissue were pooled together.

#### 2.4.9 qPCR for identification of MAP DNA from tissue and fecal samples

To detect MAP genomes in DNA samples, a two-duplex real-time qPCR assay targeting IS900 and F57 was employed (Slana et al., 2008). Multiple copies of IS900 serve as sensitivity indicators, while single copies of F57 confirm the assay's specificity. The internal amplification control (IAC) is used to monitor the overall performance of the assay and to ensure that the amplification process is working properly. The IAC plasmid is multiplied alongside the target genes using the same primers, allowing for a direct comparison of the performance of the assay between samples. The optimized mixture for both assays is comprised of 10 μl of TaqMan Fast Advanced Master Mix, 10 pmol of primers (IS900Forward or F57Forward and IS900Reverse or F57Reverse), 1 pmol each of the FAM-labeled IS900qPCR or F57qPCR probe and the Cy5-labeled IACqPCR probe, 0.2 U of Uracil DNA Glycosylase, 50 copies of the IS900 or F57 internal amplification control plasmids, and 4 μl of DNA sample, making a total volume of 20 μl (Slana et al., 2008). The amplification and fluorescence detection are performed using a Bio-Rad CFX96 thermocycler and 96-well PCR plates. The protocol includes an initial 10 minutes at 37°C, followed by a 15 minute denaturation at 95°C, and 47 cycles of 5 seconds at 95°C and 40 seconds at 60°C (Slana et al., 2008).

### 2.5 *Ex-vivo* trial to evaluate the functionality of effector immune cells in killing wild-type MAP

#### 2.5.1 Preparation of antigen presenting cells to prime effector immune cells

The *ex vivo* trial was designed and conducted as previously described (Abdellrazeq et al., 2018) (**Figure 1D**). Blood samples were drawn from calf vaccinated and control groups at 15 weeks after inoculation to create dendritic cells (DC) from monocytes) for antigen presentation to monocyte-depleted PBMCs (md-PBMCs) isolated at 16 weeks post-inoculation. As directed by the manufacturer (Miltenyi Biotec Inc.), magnetic microbeads covered with anti-human CD14 mAb were utilized to collect monocytes. The monocytes (2x10^6^)/well were seeded in wells of six-well plates and cultured in a nutrient-rich culture medium (RPMI1640 medium supplemented with 10% calf serum, 2 mM mercaptoethanol, 1 mM glutamine, 100 unit/ml penicillin G, and 100 μg/ml streptomycin sulphate) and a DC growth stimulant mix of GM-CSF and IL-4. On day 3, fresh medium containing the cocktail was added to the cell cultures, replacing 1.4 ml of the old medium. On day 6, monocyte-derived dendritic cells were exposed to 5x10^6^ MAP cells per well. The MAP strain for stimulation matched the strain of inoculation in each group. The dendritic cells that were activated were incubated with MAP (antigen) for 3 hours at a 37°C incubator with 5% CO2. After this, the cells were washed three times with culture medium to eliminate any remaining unbound bacteria.

Fresh md-PBMCs (2x10^6^) were prepared at 16 weeks post-inoculation. The md-PBMCs were added to their autologous stimulated dendritic cells. After six days of culturing the cells together, they were collected and utilized in an assay to measure the viability of the MAP antigen (Abdellrazeq et al., 2018).

#### 2.5.2 Preparation of macrophages for use as a target cell for MAP infection

The monocytes isolated from PBMCs at 16 weeks post-inoculation were used for generating monocyte-derived macrophages. CD14 magnetic microbeads were used for monocyte isolation as described. The monocytes were resuspended in a complete medium and cultured in 6 well plates overnight. The next day, the cells that did not attach to the culture surface were eliminated. Fresh medium was added to the adherent cell cultures. On day 4, cells were harvested, counted, and 2×10^6^cells/well were cultured for two more days. On day 6, monocyte-derived macrophages were incubated with wild-type MAP at MOI of 10:1 (2x10^7^ MAP). The plates were incubated for 3 hours at 37° C incubator with 5% CO2, after centrifugation 700 ×g for 5 minutes. The cells were washed five times with culture medium to eliminate any remaining unbound bacteria. The macrophages that had attached to the culture surface and were infected were collected for the MAP viability assay.

#### 2.5.3 Evaluation of MAP viability in macrophages after exposure to primed and up-primed effector immune cells

The unprimed md-PBMC and mdPBMC pulsed with monocyte-derived dendritic cells were incubated with infected and uninfected monocyte-derived macrophages for 6 hours at 37°C, 5% CO2. Control tubes were prepared as follows: each tube contained 2x10 (7 )bacteria including fully live, half live, and fully killed. The bacteria were heat-killed at 90 °C for 15 minutes. A second set of controls were 2x10^6^macrophages incubated with 100% live, 50% live, and 100% killed MAP (MOI=10). After incubation, non-adherent cells were washed, and the adherent cells were harvested and prepared for MAP viability assay as previously described (Abdellrazeq et al., 2018). Harvested cells were broken down by mixing them with 2 ml of a 0.01% saponin solution and were left to react at 37°C for 15 minutes. After centrifuging at 4500 ×g for 30 minutes., the pellet was washed with 1 ml of water. The harvested washed bacteria were resuspended in 400 μl of water. The DNA-binding dye propidium monoazide (PMA), 1μl of 20 mM, was added to the previously prepared bacterial suspension and incubated for 5 minutes on a rocker at room temperature. PMA binds to DNA of dead cells with high affinity After incubation, cells were exposed to light in A PMA-Lit^TM^ LED Photolysis Device (Biotium, USA) for 10 minutes. During light exposure the covalent reaction of dye with DNA results in permanent modification of DNA. Cells were then harvested for DNA extraction after centrifugation at 1000xg for 5 minutes. The DNA was obtained by using the DNeasy® Blood and Tissue kit (Qiagen, USA), and the procedure was carried out as instructed for bacteria classified as Gram-positive. All DNA samples were normalized by being diluted to a concentration of 1 ng/μl and qPCR was carried out to target F57 and quantify the number of live MAP cells as explained above.

### 2.6 Statistical analysis

All statistical analysis were conducted using prism software *ver*. 9.2.0 (GraphPad Software, San Diego, California). The difference in the numbers of animals that are positive for MAP (Mycobacterium avium subsp. paratuberculosis) culture or tissue between groups were analyzed by chi-square test and one-way ANOVA. The results of mice cytokine secretion and calf gene expression were analyzed using one-way ANOVA with Bonferroni correction. The comparison of means between groups were performed employing Tukey-Kramer *post hoc* test. For comparison of survival rate of MAP in ex-vivo trial, the Ct values were normalized by transforming to the percentage of live cells, and the results were analyzed performing one-way ANOVA and Tukey-Kramer tests. The PCA analysis of gene expression in calf model was performed using R studio *ver*. 1.4.1717 (Boston, MA). A P value of less than 0.05 indicates statistical significance in the results.

## 3 Results

### 3.1 Confirmation of *BacA* and *IcL* transductants

Essential MAP genes were removed from the MAP genome using specialized transducing mycobacteriophages. Colonies were grown on 7H11 plates with hygromycin for both Δ*BacA* and Δ*IcL*. The hygromycin resistance cassette could be amplified from all the screened putative *IcL* mutant colonies screened (**Figure 2**; primer set 1), while only 1 out of the 3 obtained putative *BacA* mutant colonies screened allowed the same. Δ*BacA* and Δ*IcL* were evaluated for the effective elimination of genes conducting the PCR assay for crossover regions. The assay used specific primers that were designed to target the crossover region between the deleted genes and the AES and MAP genome of the respective essential gene. (**Figure 2**; primer set 3). All colonies that had the hygromycin resistance cassette were checked for recombination by these primers. The one *BacA* colony checked indicated accomplished recombination, while one out of three *IcL* colonies indicated this. Wild-type MAP DNA was utilized as negative control.

### 3.2 Evaluation of host-specific essentiality of genes and immunogenicity of knockouts in mouse trial Δ*BacA* and Δ*IcL* colonization of liver and spleen in mice

**Figure 3:**
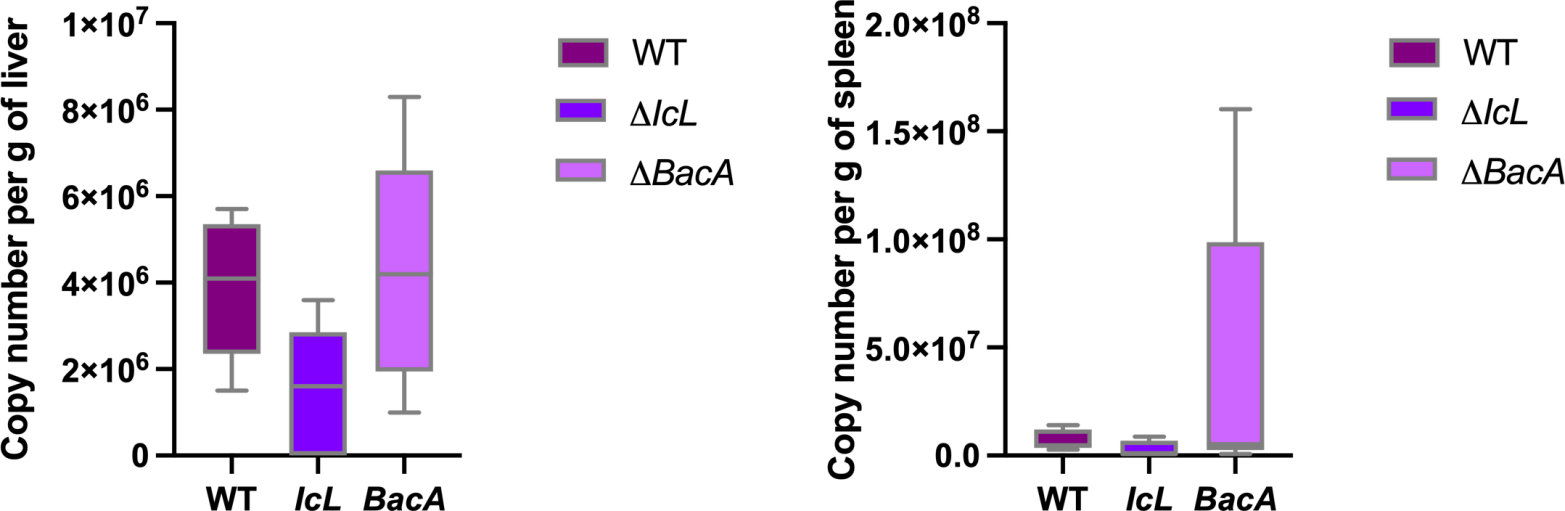
MAP quantification in mice (n=14/group) liver and spleen, three weeks after intraperitoneal infection with 5×10^8^ CFU of MAP strains. F57 qPCR was employed to quantify genomic equivalents (GE) of MAP in 1 gram of tissue. The difference among groups were assessed by one-way ANOVA and then further validated by Tukey-Kramer test.

To evaluate the ability of each strain to colonize mouse tissue, liver and spleen samples were collected from each mouse 3 weeks after inoculation. DNA was extracted from tissue and MAP F57 qPCR was performed. Both Δ*BacA* and Δ*IcL* colonized liver and spleen of the intraperitoneally infected mice, with no statistically significant difference in GE/g in liver and spleen between mice infected with Δ*BacA* and Δ*IcL* compared to positive controls with average of 2.3×10^7^, 2.2×10^6^, and 5.6×10^6^ CFU/g, respectively (**Figure 3**). Nodular appearance was observed in 40% of animals infected with wild type group, but not in any of animals infected with mutant strains.

### 3.3 Δ*BacA* induce higher secretion of pro-inflammatory cytokines in mice serum

**Figure 4:**
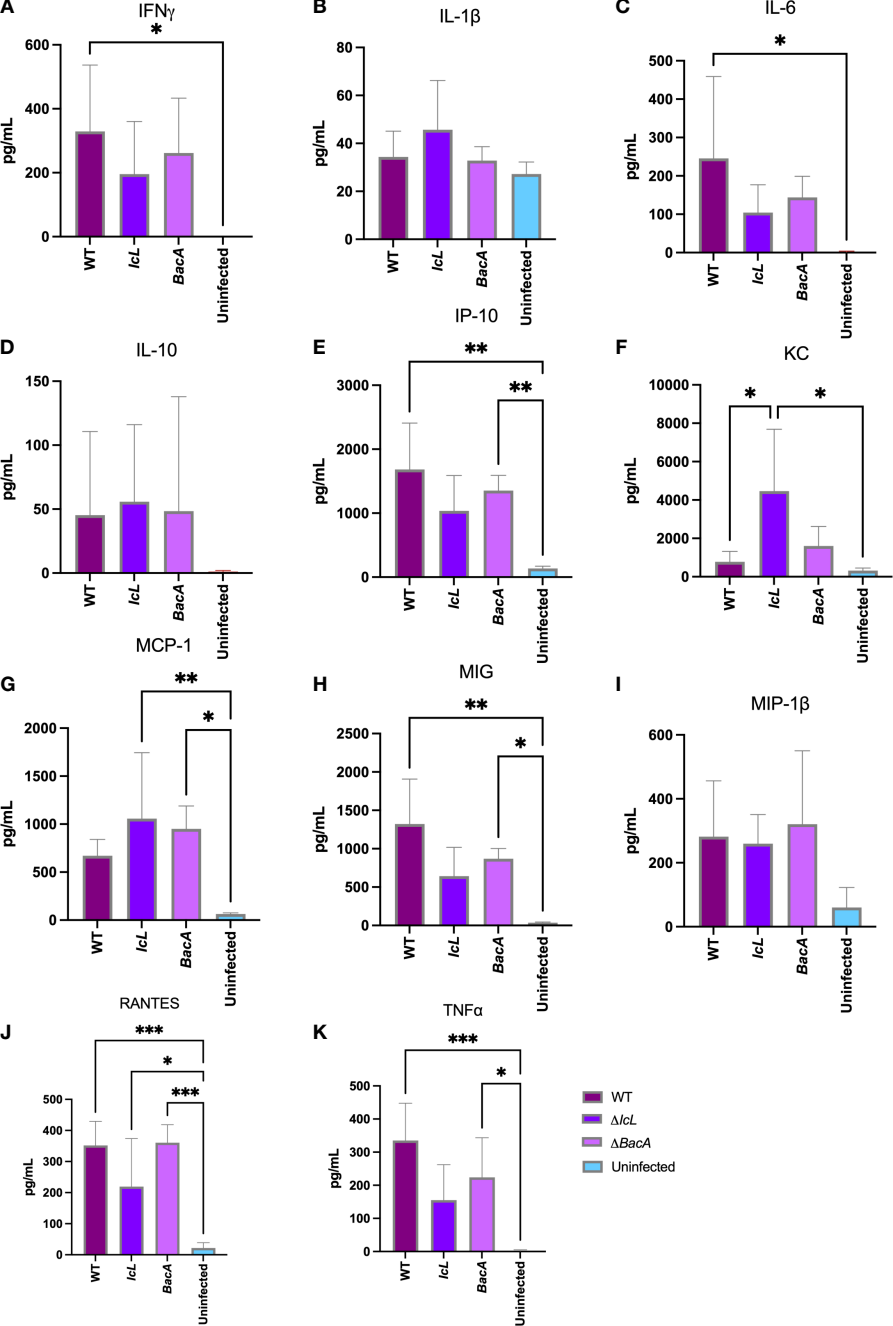
Analysis of cytokine secretion in mice (n=14/group) serum three weeks after intraperitoneal infection with 5×10^8^ CFU of MAP wild-type and mutant strains. The uninfected control received PBS. The secretion level of cytokines in the serum were measured by Luminex assay. The difference among groups were assessed by one-way ANOVA and then further validated by Tukey-Kramer test. * P≤0.05; ** P≤0.01; *** P≤0.001.

Blood samples were collected from each mouse 3 weeks after inoculation. Multiplex Luminex assay was employed to investigate the secretion of pro-inflammatory Th1 type cytokines and IL-10 in mouse serum (**Figure 4**). The secretion levels of IL-6 and IFNγ were only significantly higher in the wild-type group compared to the uninfected control (**Figure 4A, C**). The secretion levels of IP-10, TNFα, RANTES, and MIG was significantly higher in both wild-type and Δ*BacA* groups than uninfected control (**Figures 4E, H, J, K**). The secretion level of MCP-1 was higher in both mutant groups compared to uninfected control (**Figure 4G**). The secretion level of KC was higher in the Δ*IcL* group than both wild-type and uninfected controls (**Figure 4F**). Although the secretion level of anti-inflammatory cytokine, IL-10, was higher in the Δ*IcL* group, the difference was not significant between groups (**Figure 4D**).

### 3.4 Confirmation of essentiality of genes and immunogenicity of knockouts in calf trial

#### 3.4.1 Bacteria shedding in fecal matter

The MAP culture qPCR indicated that all the inoculated animals had passive MAP shedding 24 hours after inoculation. At two weeks post inoculation a fecal sample of one animal from each mutant groups and 4 animals from wild type group were MAP culture qPCR positive. At four weeks post inoculation, fecal samples from 3 animals from the Δ*BacA* group and 4 animals from each Δ*IcL* and wild type group were MAP culture positive. From 6 to 12 weeks post inoculation, fecal samples from animals from mutant and wild-type groups were either MAP culture negative or only one or two animals were positive from each group, respectively. One animal from wild type group was MAP culture positive at 16 weeks post inoculation. However, fecal samples from both Δ*BacA* and Δ*IcL* were MAP culture negative at two last time points (14 and 16 WPI) before euthanasia.

#### 3.4.2 Both ΔBacA and ΔIcL disappear from calf tissue

To evaluate the ability of each strain to colonize calf tissue, ileum, jejunum, ileocecal valve, and their associated lymph nodes were collected from each calf 4 months after inoculation. DNA was extracted from tissue directly and from bacterial culture of tissues after 49 days. MAP qPCR was performed on all DNA extracted from cultures and directly extracted from tissue. No tissue cultures were MAP positive from the Δ*BacA* and Δ*IcL* groups. Five calves from positive control group were positive for cultures of ileum lymph nodes, four animals were positive for culture of ileocecal lymph nodes, three animals were MAP positive in jejunum, two animals in ileum and ileocecal valve, and one animal was positive for MAP culture of jejunum lymph nodes. The number of culture positive animals in the wild-type group was significantly higher than mutant groups. No MAP was detected by qPCR in tissues of the Δ*BacA* and Δ*IcL* groups. However, MAP DNA was detected in ileum lymph nodes of two animals from the WT group and ileocecal valve lymph node of one animal (**Tables 2**, **3**).

**Table 2:** Number of animals positive for MAP culture.

	Ileum	Jejunum	Ileocecal valve	Ileum LN	Jejunum LN	Ileocecal valve LN
WT	2	3	2	5	1	4
Δ*IcL*	0	0	0	0	0	0
Δ*BacA*	0	0	0	0	0	0
Uninfected	0	0	0	0	0	0

Homogenized tissue was cultured by ParaJEM culture system. Forty-nine days after incubation DNA was extracted from culture broth. F57 qPCR was employed to evaluate presence or absence of MAP DNA.

**Table 3:** Number of animals with MAP qPCR positive result.

	Ileum	Jejunum	Ileocecal valve	Ileum LN	Jejunum LN	Ileocecal valve LN
WT	0	0	0	2	0	1
Δ*IcL*	0	0	0	0	0	0
Δ*BacA*	0	0	0	0	0	0
Uninfected	0	0	0	0	0	0

DNA was extracted directly from 2.5 g of tissue and F57 qPCR was employed to evaluate presence or absence of MAP DNA.

#### 3.4.3 ΔBacA induce proliferation of CD8+, CD4+, and CD4CD45RO+ cells in calves at 16 weeks post-inoculation

**Figure 5:**
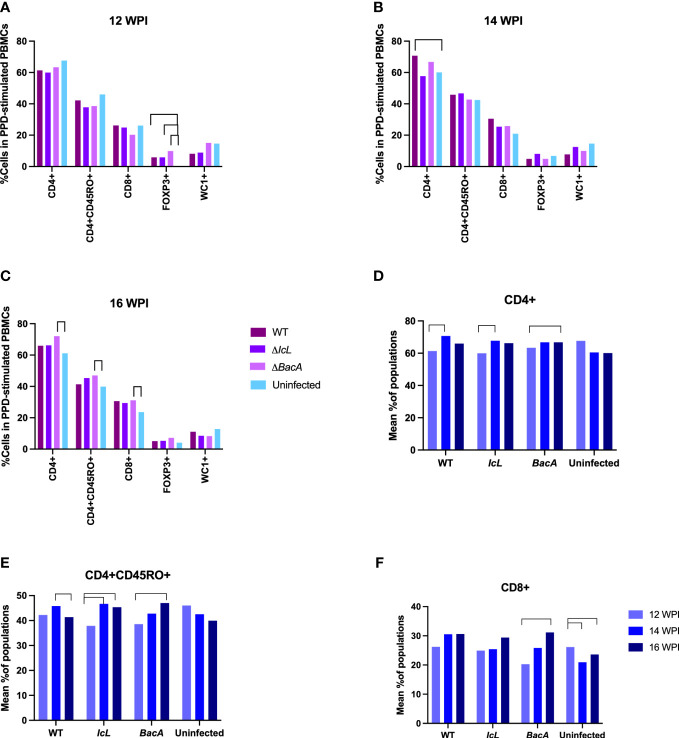
**(A-C)** Lymphocyte proliferation after stimulation of PBMCs with PPDa for 24 hours. PBMCs were isolated from all mutants and control groups at 12-, 14-, and 16- weeks post-inoculation and they were stimulated with MAP antigen for 24 hours. Flow cytometry was employed to evaluate populations of proliferated immune cells. The difference among groups were assessed by one-way ANOVA and then further validated by Tukey-Kramer test. **(D-F)** Changes in cell populations within a group over time. The difference between timepoints were evaluated by one-way ANOVA followed by Tukey-Kramer test. Significant difference between groups (P value> 0.05) is indicated by lines above bars.

PBMCs from all treatment and control groups were analyzed by flow cytometry after stimulation with PPDa at 12-, 14- and 16-weeks post-inoculation, to evaluate the proliferation of CD4+, CD4+CD45RO+, CD8+, FOXP3+ and WC1+ T cell populations. Although both Δ*BacA* and Δ*IcL* groups displayed significant increases in CD4+ and CD4+CD45RO+ populations over time, only Δ*BacA* induced an increase in the CD8+ population over time and a decrease in the FOXP3+ population at 14 weeks post-inoculation (**Figures 5E, F, H**). All inoculated groups indicated a significantly lower population of activated T helper cells (CD4+CD45+) at 12 WPI in agreement with a significantly higher population of FOXP3+ cells at 12 weeks post-inoculation (**Figure 5A**). Only Δ*BacA* induced a significantly higher population of CD4+, CD4+CD45+, and CD8+ cells compared to the uninfected control at 16 weeks post-inoculation (**Figure 5C**). The group inoculated with wild type also had a significantly higher percentage of CD4+ cells compared to the uninfected control at 14 weeks post-inoculation (**Figure 5B**). However, no meaningful variation was discovered between wild-type and mutant strains for immune cell populations.

#### 3.4.4 ΔBacA induce higher transcription of proinflammatory cytokines compared to ΔIcL and wild-type

**Figure 6:**
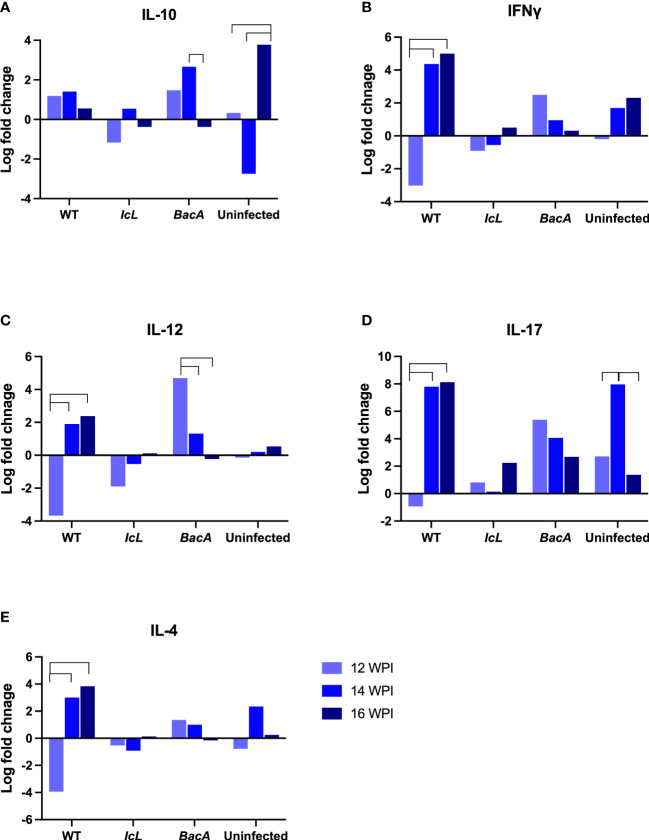
Cytokine expression within each group after stimulation of PBMCs with PPDa at 12-, 14-, and 16-weeks post inoculation. The difference among groups were assessed by one-way ANOVA and then further validated by Tukey-Kramer test.

**Figure 7:**
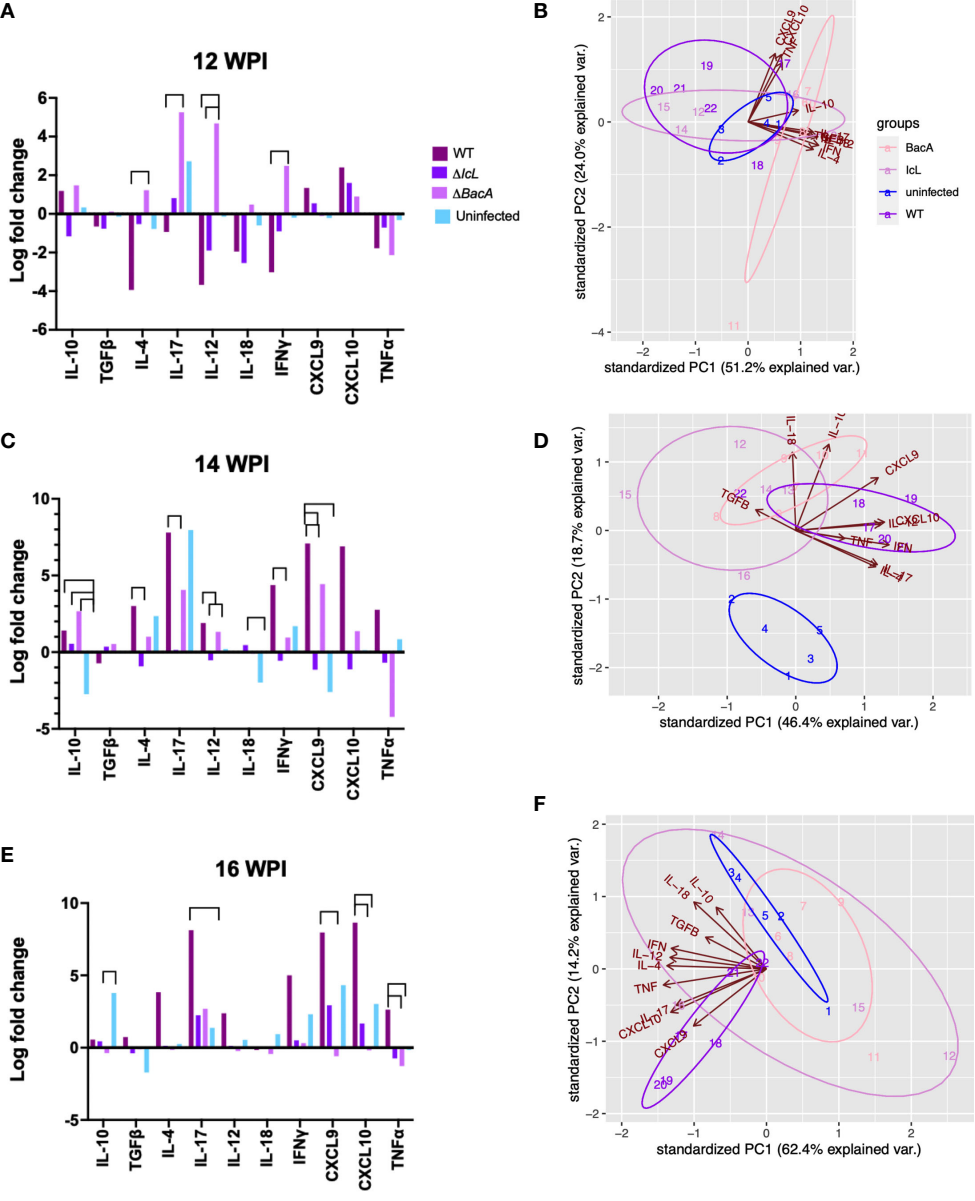
Cytokine expression analysis by RT-PCR, comparing groups at different timepoints. PBMCs were isolated from different groups (Uninfected =5, BacA=6, IcL=6, and wild-type=6). After 24 hours of in vitro stimulation with PPDa, RNA was extracted from PBMCs and reverse transcribed. RT-PCR was performed on cDNA, using 18s rRNA as a housekeeping gene and unstimulated samples as a calibrator. The data was analyzed by ΔΔct and the difference among groups were assessed by one-way ANOVA and then further validated by Tukey-Kramer test. **(B, D, F)** Groups are color coded and numbers indicate animal IDs. Each group is clustered with a different ellipse. The vectors of the loading plots showing how strongly each variable influence a principal component, with their directions indicating how different variables are correlated with one another. The smaller the angel between two vectors, the more positively correlated they are. If they diverge and form a large angel close to 180 degrees, they are negative correlated.

Real-time PCR was employed to evaluate the relative changes in cytokine transcription of PPDa-stimulated PBMCs at 12-, 14- and 16-weeks post-inoculation to determine the MAP specific recall response. To compare the level of gene transcription in PBMCs after stimulation with MAP antigen (PPDa), the non-stimulated cells were used as a calibrator and all the upregulation and downregulation of the genes in stimulated cells were compared to the non-stimulated homolog of the same sample. Pro-inflammatory cytokines including IL-12, IL-17, IL-18, IFNγ, CXCL9, CXCL10, and TNFα, and anti-inflammatory cytokines including IL-4, IL-10, and TFGβ were studied. The transcription of IFNγ and IL-17 were upregulated in all timepoints in the Δ*BacA* group (**Figures 6C, D**), and they were transcribed significantly higher than the wild-type group at earlier timepoint at 12 WPI (**Figure 7A**). The transcription of these two cytokines also increased significantly over time in the wild-type group (**Figures 6C, D**). The transcription of IL-12 in the Δ*BacA* group was upregulated at 12- and 14-weeks post-inoculation and it was significantly higher than wild-type and Δ*IcL* groups at 12- and 14-weeks post inoculation, respectively (**Figures 7A, B**). However, it indicated a significant decrease over timepoints (**Figure 6B**). At 14 weeks post-inoculation both wild-type and Δ*BacA* groups had significantly higher transcription of CXCL9 in response to PPDa stimulation compared to uninfected control, and wild-type was higher than Δ*IcL* and Δ*BacA* groups at 14- and 16-weeks post-inoculation, respectively (**Figures 7B, C**). The wild-type group displayed a higher transcription of CXCL10 and TNFα compared to vaccinated groups at 16 WPI.

With respect to the transcription of anti-inflammatory cytokines in response to MAP antigen, the transcription of IL-10 was upregulated in wild type group at all timepoints (**Figures 6A**). In contrast, IL-10 was downregulated and decreased significantly in the Δ*BacA* group at 16 weeks post inoculation, and the expression of IL-10 was significantly lower than uninfected control (**Figure 7C**). However, no significant difference was evident in transcription level of TGFβ between groups at different timepoints and within groups over time. The transcription level of IL-4 significantly increased in wild type group at later timepoints (**Figure 6E**). The PCA analysis indicated that the variation between individual animals in the Δ*IcL* group was high at all timepoints as they were less tightly clustered (**Figures 7B, D, F**). This suggests that Δ*IcL* induces different immune responses and activation levels in individual animals. The PCA analysis also indicates a positive correlation between CXCL9, CXCL10, and TNFα , and between IL-4, IL-12, IL-18, IL-17, and IFNγ at 12 weeks post inoculation as the direction of their vectors are matching. Positive correlation between IL-17 and IL-4 and between CXCL-10 and IL-17 is also indicated at 14- and 16-weeks post inoculation, respectively.

### 3.5 Macrophages from vaccinated groups, alone or incubated with unprimed PBMCs, have better MAP killing activities than their homologs from wild-type group

**Figure 8:**
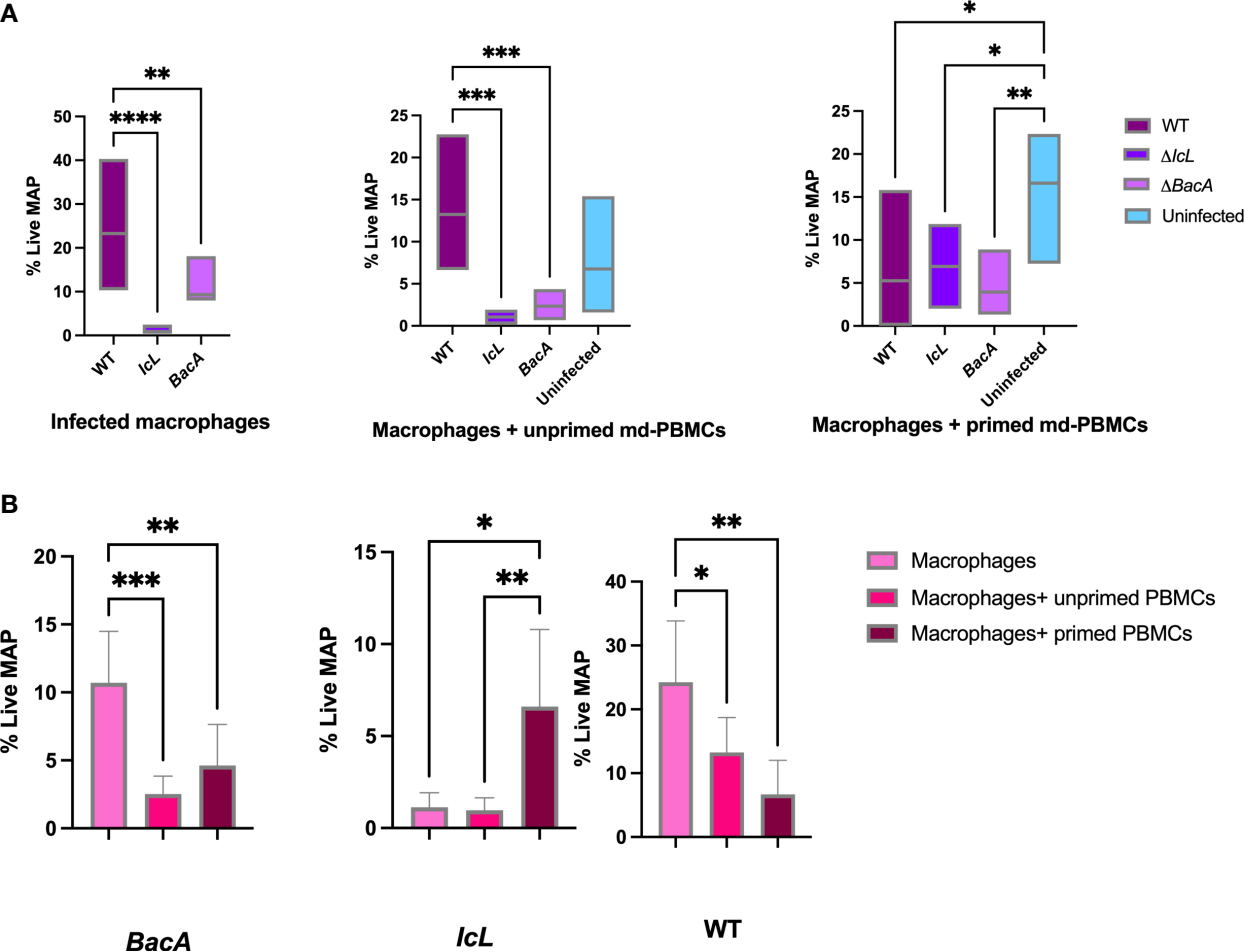
**(A)** MAP viability assay comparing percentage of live MAP in infected macrophages alone, infected macrophages incubated with unprimed md-PBMCs, and infected macrophages incubated with primed md-PBMCs. **(B)** Viability assay comparing different treatments within groups. The difference among groups were assessed by one-way ANOVA and then further validated by Tukey-Kramer test. * P≤0.05; ** P≤0.01; *** P≤0.001.

A bacterial viability assay was employed to evaluate the killing activity of macrophages alone, macrophages incubated with primed PBMCs, and macrophages incubated with unprimed PBMCs. The survival rate of wild-type MAP in infected macrophages alone, and infected macrophages incubated with unprimed PBMCs was significantly higher in the wild-type group compared to the vaccinated groups (**Figure 8A**). However, there was no significant difference between treatment groups when the macrophages were incubated with primed PBMCs. This suggests that the proliferation and functionality of immune cells after the recall response was similar in all treatment groups. In both Δ*BacA* and wild-type groups the survival rate of MAP in macrophages alone was significantly higher than macrophages incubated with PBMCs, regardless of PBMCs being primed (**Figure 8B**), suggesting that effector T cells contribute to the killing of MAP. However, the result of the Δ*IcL* group was unexpected as the killing activity of macrophages alone was higher compared to macrophages incubated with unprimed PBMCs (**Figure 8B**). The data from infected macrophages alone from the uninfected control were eliminated from the analysis due to contamination of the cell culture.

## 4 Discussion

The present study was designed to evaluate the *in vivo* survival of two knockout live attenuated strains of *Mycobacterium avium* subsp. *paratuberculosis* and their efficacy to induce cell-mediated immune responses by a comparative approach in mouse and natural host calf models. A previous study identified genes necessary for *MAP* survival in dairy calves and permitted us to select a gene to knockout that is essential for MAP survival *in vivo* (MAP1643, *IcL*) or one which is required for intracellular growth (MAP1531c, *BacA*) (EshraghiSamani et al., 2022). The results of the current study confirmed the attenuation of these mutants in calves and indicated that disruption of these genes affect MAP's intracellular survival without affecting its immunogenicity. The complementation of either genes is expected to restore the virulence.

Mouse and calf infection trials were employed to evaluate the immunogenicity of two vaccine candidates, confirm the attenuation in calf, and assess the species-specific essentiality of candidate genes. Knowing these genes are identified to be essential for MAP survival in natural host, a mouse model was employed to evaluate their role in MAP survival in this common laboratory animal. The inoculation of calves happened through the oral route, while in the mouse model animals were inoculated intraperitoneally. The results revealed that despite attenuation of mutants in calf model, there was no significant difference in bacterial load of mutants or wild-type in liver and spleen of infected mice. Although nodular lesions were seen at the necropsy in 40% of the mice infected with wild-type, detailed gross pathology examination revealed no lesions in the mutant-infected animals. A rationale framework to study *MAP* vaccines has been proposed by the multi-institutional USDA-funded research consortium (the Johne's disease integrated program (JDIP)) and in the previous studies (Bannantine et al., 2014a), recommending to carry out a three-stage trial protocol, utilizing a sequential process starting with macrophages, followed by testing in mice and finally in the intended natural host. This is mostly to evaluate the attenuation of different vaccine candidates in macrophage and mouse models to narrow down the options for *in vivo* trials in more challenging ruminant models. Although some researchers stuck to this framework, some vaccine research was limited to only one or two models mostly including *in vitro* macrophage trials and mouse trials. Macrophage model has shown to be reliable predictor of mycobacterial infections (Juste et al., 2016b; Tanner et al., 2020; Bok et al., 2021). However, the data from this study and investigations by other researchers raise important questions on whether mice is good predictors and representative models for the evaluation of *MAP* vaccines, and the use of the natural host model is strongly recommended.

The mouse trial results indicated that both vaccine candidates colonize tissue in comparable bacterial load with wild-type MAP. The liver and spleen of mouse had high number of MAP CFUs with no differences between groups. In contrast, calves tissue culture and qPCR indicated that interruption of both *IcL* and *BacA* genes hamper the capacity of the mutants to colonize in tissue as no bacteria were recovered from intestinal tissue and their draining lymph nodes. Considering the difference between type of tissues and route of administration, this data suggests that mouse intraperitoneal model might not be a good predictor for evaluation of MAP attenuation in its natural host. Earlier studies indicated that isocitrate lyase is required for the persistence of *Mycobacterium tuberculosis* in lungs in tail-vein injected mice (McKinney et al., 2000). It has also been indicated that *BacA* mutants of *Mycobacterium tuberculosis* lose their capacity to maintain chronic persistence in tissue of murine model infected *via* aerosols (Domenech et al., 2009). In consideration of the appropriateness of mouse models of paratuberculosis, it is first important to realize the large difference exist between gastrointestinal tract of bovine and rodents. It has proven difficult to establish a MAP infection through an oral route in mice or to generate clinical signs and intestinal lesions. Species-specific attenuation of mutant strains is consistent with previous literature (Scandurra et al., 2010; Bannantine et al., 2014b) where evaluation of MAP 1566 live-attenuated vaccine strain in mouse model indicated some level of protection against challenge, while the evaluation of same vaccine strains was unable to show promising results in goat model (Bannantine et al., 2014b). In present study, the opposite is observed where vaccine strains were not attenuated for colonization in mice tissue, while they were both attenuated for colonization in natural host. So, the result of laboratory models should be interpreted with more caution, and it is ideal to employ natural host models at final stages of vaccine development to fully evaluate vaccine candidates.

The disappearance of mutants from calf tissue was in agreement with the results of an *ex-vivo* viability assay, where wild-type MAP survival was also hindered by effector immune responses in bovine macrophages alone and macrophages incubated with unprimed PBMCs in both vaccine groups. The difference between macrophage alone compared to macrophage incubated with primed/unprimed PBMCs in wild-type and Δ*BacA* groups suggests that Δ*BacA* induce adaptive immune responses that are efficient in not only clearing the *mutant* strain from tissue but also decrease the survival of wild-type MAP in macrophages. The responsiveness of immune cells in *ex-vivo* assay was in accordance with higher populations of CD4+, CD8+, and CD4+CD45RO+ cells at 16 weeks post-inoculation in the Δ*BacA* group compared to uninfected control after exposing PBMCs to MAP PPD antigen. For vaccine induced protection against mycobacterial infections, CD4+ cells are vital and multiple studies have demonstrate that CD8+ cells play critical role in controlling mycobacterial infections *via* perforin, granzymes, and granulysin (Sakai et al., 2014; Lin and Flynn, 2015). There was no meaningful difference in the recall response among vaccine and wild-type groups after exposing macrophages to primed PBMCs. This suggests that after being primed, cytotoxic T-cells are able to expand at the same level in mutants and wild-type groups in recall response. These findings also suggest that only evaluating the proliferative response of PBMCs toward MAP antigen does not clearly predict the functional capacity of CD4+ and CD8+ populations against MAP.The *ex-vivo* result of the Δ*IcL* group is more difficult to interpret as the primed PBMCs indicated higher survival rate for MAP compared to macrophage alone or incubated with unprimed PBMCs. Although deletion of ABC transporter and isocitrate lyase hinders the capacity of mutant to persist in calf tissue evaluated at the end point, it does not change their ability to induce immune responses compatible with wild-type strain. The clearance of mutants from calf tissue at the end point (18 WPI) was in accordance with disappearance of mutants from feces at timepoints closer to the euthanasia (14 and 16 WPI). In the natural setting, animals are always re-exposed to MAP, from the outside environment and at the body microenvironment level from neighboring or more sites. So, memory responses are always activated at re-exposure. Unless the chronic infection induces anergy and exhaustion at clinical stages. First and foremost, the live attenuated vaccines in the present study are aimed to be evaluated as a preventive measure. Further investigations and trials are required to evaluate their therapeutic benefits in infected animals.

Both vaccines and wild-type strains indicated similar proliferative responses to PPDa antigen and there was no significant difference between treatment groups at all three time points. However, all treatment groups had a higher population of FOXP3+ T-cells compared to the uninfected control at 12 weeks post inoculation. This suggests that the inhibitory-regulatory responses start at earlier timepoint in all treatment groups. The early proliferation of Treg cells can be explained by the aberrant production of pro-inflammatory cytokines at earlier stages of infection which can lead to inhibitory immune responses to protect the host. These findings agree with those of previous studies where the Treg cells are more dominant at earlier stages of tuberculosis infection compared to latent stage (Cardona and Cardona, 2019). The higher population of Treg cells was not maintained throughout later timepoints, which was in accordance with higher population of CD4+ T-cells in wildtype group and CD4+, CD8+, and CD4+45RO+ T-cells in the Δ*BacA* group compared to uninfected control at 14- and 16-weeks post inoculation, respectively. The higher population of T helper cells and cytotoxic T cells might be contributing to clearing the infection in the Δ*BacA* group. Although there used to be a dichotomy paradigm of having a protective proinflammatory response at early stages and then a shift to anti-inflammatory responses at later stage, recent research indicated that there is no such distinction in immune reopposes to mycobacterial or other intracellular infections and there is cross-regulation of pro and anti-inflammatory responses at different stages (Stabel and Bannantine, 2019). This parallel activation of pro- and anti-inflammatory responses in infected animals was also evident in our study, where there was an upregulation of both pro- and anti-inflammatory cytokines including IL-10 and interferon gamma induced cytokines, respectively.

Evaluation of cytokines secretion in mice model indicated that although only three weeks passed after inoculation with wild-type MAP, almost all the proinflammatory cytokines including IFNγ, IP-10, IL-6, KC, MCP-1, MIG, MIP-1β, RANTES, TNFα had concentrations higher than 200 pg/ml in serum. Gene expression analysis on stimulated calf PBMCs also indicated that although the expression of IL-10 was upregulated and the expression of IFNγ was downregulated at 12 weeks post-inoculation, both IFNγ-induced cytokines CXCL9 and CXCL10 were upregulated. At both 14- and 16-weeks post-inoculation, IL-4 and IL-10 were upregulated in wild-type group, while IL17, IFNγ, CXCL9, CXCL10, and TNF were also upregulated. This agreed with a high population of CD4+ cells compared to the uninfected control at 14 weeks post-inoculation. The immune type conversion paradigm has recently been the subject of some controversy as infected animals may have a substantial overlap between type 1 and type 2 immunity, as evidenced by high levels of IL-12 and IL-18 secretions when clinical cow PBMCs are stimulated with antigen (Stabel and Bannantine, 2019). In studies of another intracellular pathogen, *Leishmania*, type 1 and 2 responses are cross-regulated and the absence of effector memory cells results in infection establishment (Carneiro et al., 2020).

It is important to wonder that if the mouse model is not able to predict persistence of bacteria in tissue, what conclusions can be drawn from the induced immune responses in the mouse model. Having only one time point in mouse trial makes the judgment more difficult. Analysis of cytokine secretion from mice indicated that absence of both genes could not change the capability of mutants to induce secretion of proinflammatory cytokines, leading to expansion of Th-1 type cell subpopulations. Data from the *in vivo* mice trial revealed that although the secretion level of IFNγ was only higher in wild-type group compared to uninfected group, proinflammatory cytokines in the Δ*BacA* group were more comparable to wild-type group as both wild-type and Δ*BacA* groups had higher secretion of IP-10, MIG, and TNFα compared to uninfected groups. IP-10 has found to be correlated with CD8+ T cell and Th1 cell responses, besides its role in cell migration (Purdie et al., 2022). It has been indicated that IP-10 secretion can also be used as a biomarker for tuberculosis (Qiu et al., 2019) and both IP-10 and MIG cytokines are both known to be upregulated in response to infection with Mycobacterium tuberculosis (Yang et al., 2014). Inoculation with Δ*IcL* also induced secretion of some proinflammatory cytokines including KC which regulates leukocyte migration and is mostly involved in repair and healing, contributing to clearance of bacteria at early stages of infection. Once MAP gets inside the macrophage, different cytokines and signaling pathways result in activation of neighboring macrophages in the microenvironment. The conditions inside the phagosome of activated macrophages will shift towards glycolysis, which convert glucose mostly to primary substrates of Krebs cycle in anaerobic conditions of activated macrophage, macrophages use up large amount of glucose in short time after activation and then the source of energy will change to glycerol (Kolliniati et al., 2022). The need of *IcL* inside activated macrophage suggests that the loss of capacity to survive and clearance of *IcL* mutants might start as early as macrophages get activated at the site of infection (McKinney et al., 2000). That justifies the need for neutrophils and monocyte/macrophages to migrate to the site of infection.

Analysis of cytokine gene expression by RT-PCR in the calf model indicated that the expressions of proinflammatory cytokines were mostly upregulated and higher than the wild-type group in the Δ*BacA* group at 12 weeks post-inoculation. However, this stage seems to be followed by an upregulation of anti-inflammatory cytokine, IL-10, at 14 weeks post-inoculation. The high expression of IL-10 in the Δ*BacA* group was in accordance with the downregulation of TNFα at the same timepoint. However, the expression of IL-10 decreased and was downregulated at 16 weeks post-inoculation in accordance with an increase in proliferation of T-cells at the same time. The expression of some cytokines was also induced by PPDa stimulation in the Δ*IcL* group including IL-17, CXCL9, and CXCL10. However, inoculation with Δ*IcL* had heterogenous effects on animals, evident by very different levels of gene expression by individuals in this group and being less tightly clustered by PCA analysis.

In summary, it can be concluded that the inoculation with Δ*BacA* strain induces the upregulation of proinflammatory cytokines earlier as it is less virulent and more vulnerable compared to wild-type MAP. Next, an upregulation of anti-inflammatory cytokines, meant to protect the host, triggers a round of cross-regulation between pro-and anti-inflammatory cytokines and the T- cell proliferation, resulting in clearance of Δ*BacA* from tissue at later timepoints, as indicated in the *ex-vivo* trial and the tissue burden. Δ*BacA* is less virulent due to its lack of ABC transporter necessary for importing hydrophilic compounds like B12. The wild-type is able to advert immune system by having all these properties and this will delay the activation of macrophages and the downstream signaling pathways (Arsenault et al., 2014)and delay the proinflammatory responses. In addition, the deletion of *BacA* has been found to impair MAP growth *in vivo* (EshraghiSamani et al., 2022), these might suggest that Δ*BacA* is not over-attenuated to get cleared before inducing immune responses, and it is not under-attenuated to persist in tissue and maintain a chronic infection.

In conclusion, examination of MAP mutants’ persistence in mouse model did not predict their persistence in the natural host. Both vaccine candidates were cleared from calf tissue and capable of inducing killing activity in immune cells. However, Δ*BacA* elicited immune responses either similar to wild-type strain or significantly higher and its effect is more homogenous compared to Δ*IcL*. There is a fine balance between Δ*BacA* attenuation and immunogenicity. The higher expression of IFNγ at earlier timepoint and higher populations of CD4+ and CD8+ cells are major contributors to clearance of Δ*BacA* from tissue. The efficacy of vaccination with the *BacA* mutant against infection with wild-type MAP remains to be investigated. In the future calf trial, the capability of *BacA* vaccine to prevent wild-type MAP colonization in tissue and fecal shedding will be evaluated.

## Data availability statement

The raw data supporting the conclusions of this article will be made available by the authors, without undue reservation.

## Ethics statement

The animal study was reviewed and approved by The calf and mouse study were reviewed and approved by the Veterinary Science Animal Care Committee of the University of Calgary, protocols AC18-0059 and AC17-0220, respectively.

## Author contributions

RE carried out the calf infection trial, analyzed the data, and took the lead in writing the manuscript with input from all authors. RA carried out the mice trial. LL created mutant strains. JB acquired funds, devised the project and the main conceptual ideas and the manuscript outline. All authors contributed to the article and approved the submitted version.
